# Dataset representing the effect of indirect band gap region of Cd-free AlGaAs buffer layer in Cu(In,Ga)Se photovoltaic cell

**DOI:** 10.1016/j.dib.2017.08.017

**Published:** 2017-08-31

**Authors:** Sadia Islam Shachi, Ali Newaz Bahar

**Affiliations:** Department of Information and Communication Technology (ICT), Mawlana Bhashani Science and Technology University (MBSTU), Tangail-1902, Bangladesh

**Keywords:** Numerical modeling, Indirect band gap region, AlGaAs buffer layer, CIGS

## Abstract

The dataset of physical properties for the proposed CIGS solar cell with Cd-free AlGaAs buffer layer has been depicted in this data article. The cell performance outcome due to different AlGaAs buffer layer band gap is reported along with optimum solar cell performance parameters for instance, open circuit voltage ${(V}_{\mathrm{oc}})$, short circuit current density ($J_{\mathrm{sc}})$, fill factor (FF), efficiency (η), as well as collection efficiency ${(\eta }_{c})$.

**Specifications Table**

**Table:** 

Subject area	*Renewable energy*
More specific subject area	*Solar cell simulation and modelling*
Type of data	*Table and figure*
How data was acquired	*Material parameters for various layers of CIGS cell has been gathered from ref*[1], [2], [3], [4], [5], [6], [7], [8], [9]*and ADEPT 2.1*[10], *an 1D online simulator has been used to elicit the performance parameter data of the proposed solar cell.*
Data format	*Filtered and analyzed*
Experimental features	*A AlGaAs buffer layer based CIGS solar cell has been fabricated as ZnO:Al/i-ZnO/Al*_*0.9*_*Ga*_*0.1*_*As/CIGS/Mo/SLG. Dataset of the performance parameters are evaluated by altering doping concentration, band gap, thickness and others physical and electrical properties of the materials.*
Data accessibility	*Dataset is within the data article*

**Value of the data**

• The dataset for physical and electrical properties of various semiconductor materials used to manufacture the proposed CIGS solar cell is defined in Table 1.

**Table 1:** Dataset for material properties of proposed CIGS solar cell layers.

Parameters	ZnO:Al	i-ZnO	Al_0.90_Ga_0.10_As	Cu(In,Ga)Se_2_
Thickness, $t_{m}\left(\mathrm{µm}\right)$	0.5	0.02	0.03	2
Dielectric constant, $K_{s}$	7.8	7.8	12	13.6
Refractive index, $N_{\mathrm{dx}}$	2	2	3.15	3.67
Band gap, $E_{g}\left(\mathrm{eV}\right)$	3.3	3.3	2.60	1.21
Electron affinity, $\chi_{e}\left(\mathrm{eV}\right)$	4.6	4.6	3.74	4.21
Electron mobility, ${µ}_{n}({\mathrm{cm}}^{2}V^{-1}s^{-1})$	100	60	200	100
Hole mobility, ${µ}_{p}({\mathrm{cm}}^{2}V^{-1}s^{-1})$	30	20	40	25
Conduction band effective density of state, $N_{c}({\mathrm{cm}}^{-3})$	2.2×10^18^	2.2×10^18^	6.5×10^17^	2×10^18^
Valence band effective density of states, $N_{v}({\mathrm{cm}}^{-3})$	1.8×10^19^	1.8×10^19^	1.12×10^19^	1.6×10^19^
Donor concentration, $N_{d}({\mathrm{cm}}^{-3})$	1×10^18^	2×10^17^	1×10^19^	0
Acceptor cConcentration, $N_{a}({\mathrm{cm}}^{-3})$	0	2×10^17^	0	5×10^18^
Electron lifetime, $\tau_{n}\left(s\right)$	5×10^−8^	6×10^−8^	2×10^−8^	1×10^−8^
Hole lifetime, $\tau_{p}\left(s\right)$	5×10^−9^	4×10^−9^	6×10^−8^	5×10^−8^
• This numerical data can be utilized by scientific community to model and simulate another CIGS solar cell.
• The rationality of other simulation process and design can be associate and confirm by evaluating these datasets.
• The future simulated model of CIGS solar cell can take suggestion from the numerical values of material parameters.

## 1 Data

The input data of electrical properties and material parameters for different layers of the proposed CIGS photovoltaic solar cell have been described in Table 1. The dataset for contact parameter of the simulation process is also presented in Table 2. All these numerical values are verified from the Ref. [1], [2], [3], [4], [5], [6], [7], [8], [9]. Fig. 2 and Table 3 showed the solar cell performance variation as a result of separate value of buffer layer band gap. Fig. 3 and Table 4 disclosed the optimum value performance parameters of the improved CIGS thin film solar cell.

**Fig. 1:**
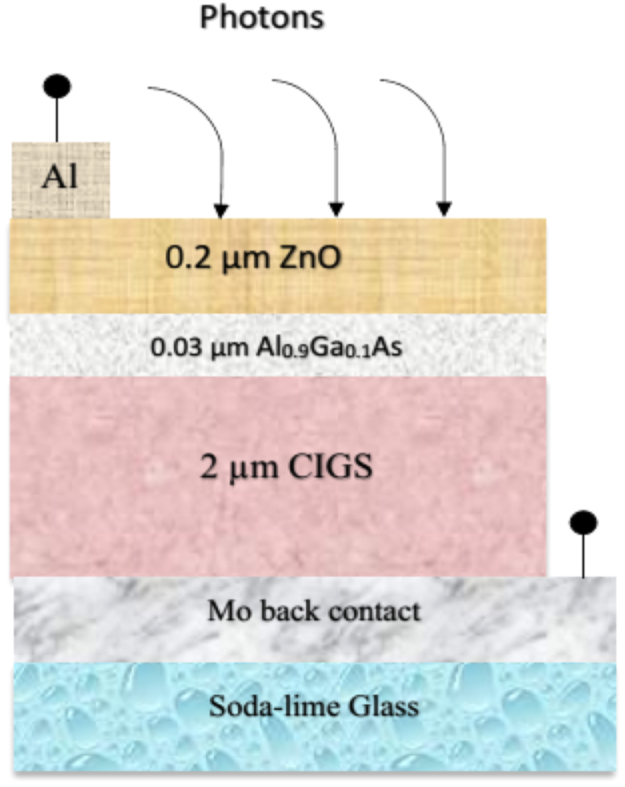
Structure of CIGS thin film solar cell.

**Fig. 2:**
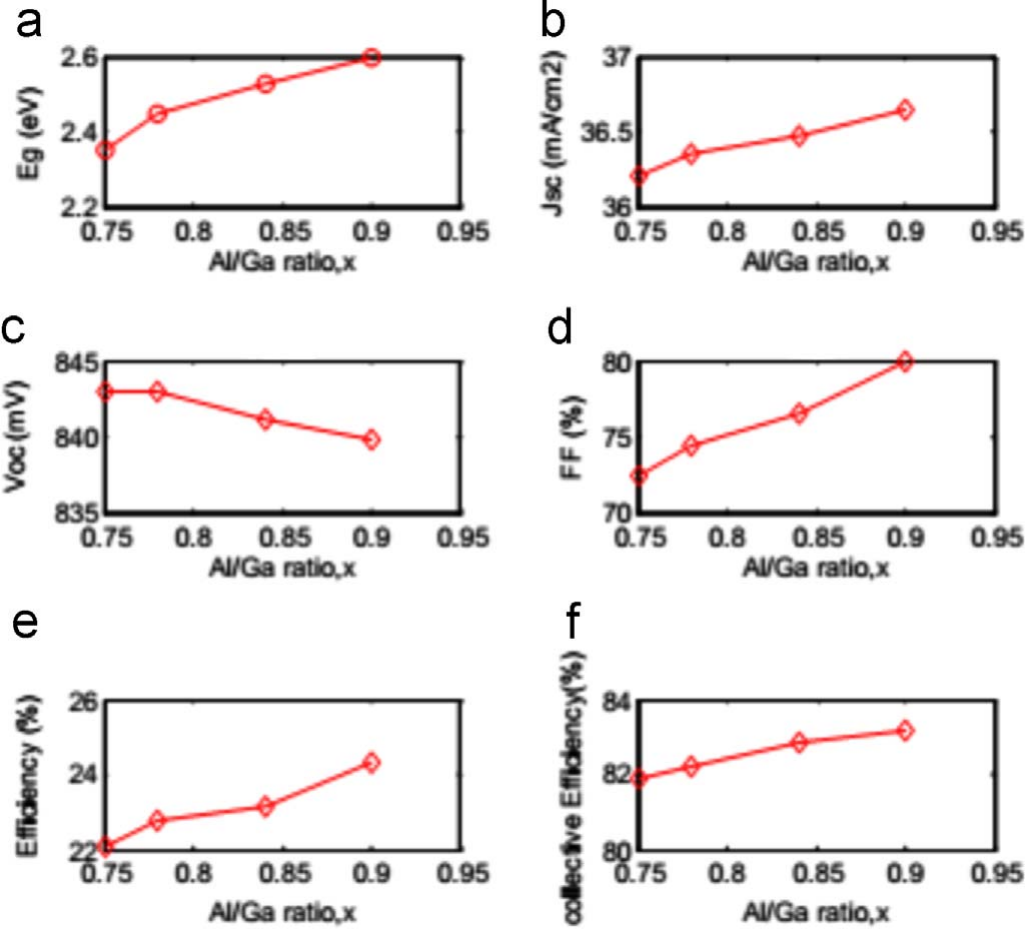
Performance analysis for various Al/Ga ratio, x.

**Fig. 3:**
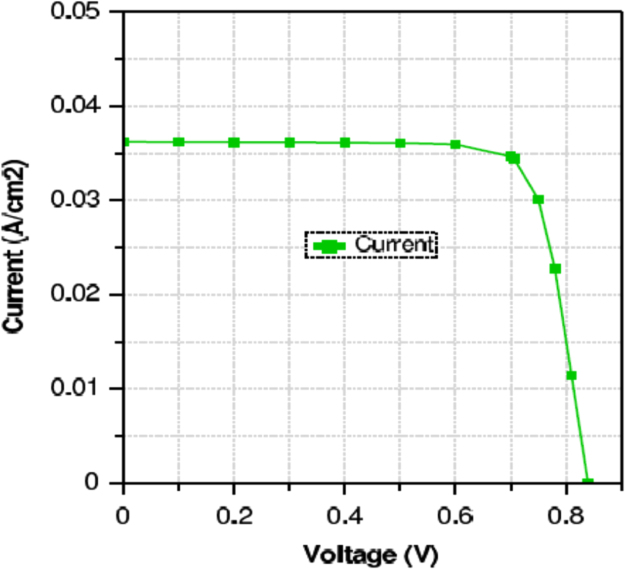
*J*–*V* characteristic curve for optimum performance.

**Table 2:** Contact parameters data used for simulation of CIGS cell.

Parameters	Front contact data	Back contact data
Reflectance	0.1	0.8
Recombination velocity for holes	10^7^	10^7^
Recombination velocity for electrons	10^7^	10^7^

**Table 3:** Efficiency analysis due to buffer layer band gap.

x	E_g_ (eV)	$J_{\mathrm{sc}}\;({\mathrm{mAcm}}^{-2})$	$V_{\mathrm{oc}}\;(\mathrm{mV})$	FF(%)	η(%)	$\eta_{c}\;(\%)$
0.90	2.60	36.21	839.76	79.96	24.32	83.16
0.84	2.50	36.21	843.15	72.56	22.16	83.14
0.78	2.45	36.20	842.96	72.41	22.09	82.15
0.75	2.35	36.21	843.02	72.39	22.10	82.56

**Table 4:** Optimum dataset of performance for simulated CIGS solar cell.

Performance parameters	Parametric value
Open circuit voltage, $V_{\mathrm{oc}}\left(\mathrm{mV}\right)$	839.76
Short circuit current density, $J_{\mathrm{sc}}({\mathrm{mAcm}}^{-2})$	36.21
Fill factor, FF(%)	79.96
Efficiency, η(%)	24.32
Collection efficiency, $\eta_{c}\left(\%\right)$	83.16

## 2 Experimental design, materials and methods

### 2.1 Device structure of proposed CIGS solar cell

The basic diagram of the proposed CIGS solar cell in the simulation study is denoted in Fig. 1. The model of the solar cell is contained ZnO:Al/i-ZnO/Al_0.9_Ga_0.1_As/CIGS materials stack on the molybdenum coated soda-lime glass substrate for more effective cell performance.

### 2.2 Effect of buffer layer band gap

ADEPT 2.1 [10], an online 1D simulator, has been used to simulate the model and investigate the effectiveness of the proposed cell. Fig. 2 depicted the cell performance with various Al/Ga ratio, *x*; a. band gap $E_{g}$, b. short circuit current density $J_{\mathrm{sc}}$, c. open circuit voltage $V_{\mathrm{oc}},$ d. fill factor FF, e. efficiency η and f. collection efficiency $\eta_{c}$.

### 2.3 Performance analysis

From the *J*–*V* characteristic curve of cell, the performance parameters such as $V_{\mathrm{oc}}$ and $J_{\mathrm{sc}}$ has been restrained as showed in Fig. 3. Also, the FF, η, and $\eta_{c}$ have been find out from the simulation output of the cell. Table 4 contains the data of all these performance parameters.
